# Mathematical Analysis of the Role of Heterogeneous Distribution of Excitable and Non-excitable Cells on Early Afterdepolarizations

**DOI:** 10.3389/fphy.2018.00117

**Published:** 2018-10-12

**Authors:** Seunghyun Kim, Daisuke Sato

**Affiliations:** 1 Department of Mathematics, University of California, Davis, Davis, CA, United States,; 2 Department of Pharmacology, University of California, Davis, Davis, CA, United States

**Keywords:** early afterdepolarizations, heterogeneity, reflection, excitable media, reentry, arrhythmias, nonexcitable gap, cardiac tissue

## Abstract

Early afterdepolarizations (EADs) are abnormal oscillations during the plateau phase of the cardiac action potential and have been linked to cardiac arrhythmias. At the cellular level, EADs can be caused by reactivation of the L-type calcium (Ca^2+^) channels, spontaneous Ca^2+^ releases from the sarcoplasmic reticulum, or both. In tissue, these EADs can trigger action potentials in neighboring cells, which may propagate as a nonlinear wave. In this scenario, EADs are attributed to cellular/subcellular/channel properties. In this study, we show a novel mechanism of EADs due to heterogeneous distribution of excitable and non-excitable cells in tissue, using a physiologically detailed computational model and mathematical analysis. In tissue, excitability of cells depends on the cell type and physiological and pathological conditions. Non-excitable cells create a non-excitable gap in tissue, which has been thought to be a cause of slow waves and reflected waves. Here, we show that the non-excitable gap also can be responsible for EAD generation. However, EADs occur only when the non-excitable gap size is optimal. If the gap size is too small, no EADs occur. If the gap size is too large, the action potential wave cannot propagate through the gap region. We also demonstrate that EADs caused by the non-excitable gap can initiate reentry in tissue, which has been linked to ventricular tachycardia and fibrillation. Thus, the non-excitable gap can lead to both focal and reentrant arrhythmias. EADs shown in this study are spatial phenomena and require tissue heterogeneity. Our study sheds light on the role of tissue heterogeneity on focal and reentrant arrhythmias.

## INTRODUCTION

Sudden cardiac death is one of the major causes of death in the world [1]. Sudden cardiac death is most often caused by arrhythmias. Under normal conditions, action potential waves propagate smoothly in the heart. During arrhythmias, in contrast, action potential waves are less organized and often show spatiotemporally chaotic behaviors. However, it is not clear how regular sinus rhythm becomes irregular arrhythmias.

Cardiac myocytes are excitable cells. These cells are coupled by gap junctions in tissue. The membrane excitability and gap junction coupling are highly heterogeneous in the heart [2, 3]. These heterogeneities are especially amplified under pathological conditions such as heart failure and myocardial infarction [4–9].

Early afterdepolarizations (EADs) are abnormal oscillations of the membrane potential during the plateau phase of the action potential. EADs can be caused by reactivation of the Ca^2+^ and/or Na^+^ channels or spontaneous Ca^2+^ releases from the sarcoplasmic reticulum, or both [10–15]. These abnormal oscillations can re-excite surrounding tissue and cause triggered activities if they overcome the source-sink mismatch [16–20].

Cardiac myocytes are electrotonically coupled via gap junctions in tissue. When some cells are excited in tissue, currents flow from excited cells to neighboring cells via gap junctions and excite the neighboring cells. The propagation excitation forms the action potential wave. Under pathological conditions such as ischemia and myocardial infarction, cells can be less excitable or non-excitable [21, 22]. If one cell is non-excitable, the membrane potential of the cell is passively changed by the membrane potential of surrounding cells. Generally, one or few non-excitable cells do not cause a problem since the action potential wave can pass through these cells. However, as the number of non-excitable cells increases, the action potential wave slows down in this region, and eventually fails to propagate when the number of non-excitable cells exceeds a certain threshold. In addition to propagation failure, non-excitable cells in tissue also can cause retrograde waves, which may lead to focal arrhythmias. This phenomenon is known as “reflection” and shown in experiments using the ventricular tissue, atrial tissue, and Purkinje fiber [23–29]. These reflected waves have been also shown in computer simulations [30–35]. The simplified mathematical model of cardiac tissue showed the mechanisms of reflected waves and the role of the non-excitable region [31]. The detailed analysis using 1- and 2-variable models has shown that the number of reflected waves is highly sensitive to the size of the non-excitable region [33, 35].

In this study, we show how non-excitable cells in excitable tissue affect EAD formation due to reactivation of the Ca^2+^ channels using computational models and mathematical analysis. The key finding in this study is that a small region (1~2 mm) of non-excitable tissue can lead to EADs and promote arrhythmias. Thus, tissue properties are critical for EAD formation as well as cellular properties. Such heterogeneous distribution of nonexcitable and excitable cells in tissue, as in pathological conditions such as ischemia, can lead to the onset of ventricular tachycardia and fibrillation.

## MATERIALS AND METHODS

### Physiological Model

**FIGURE 1 |:**
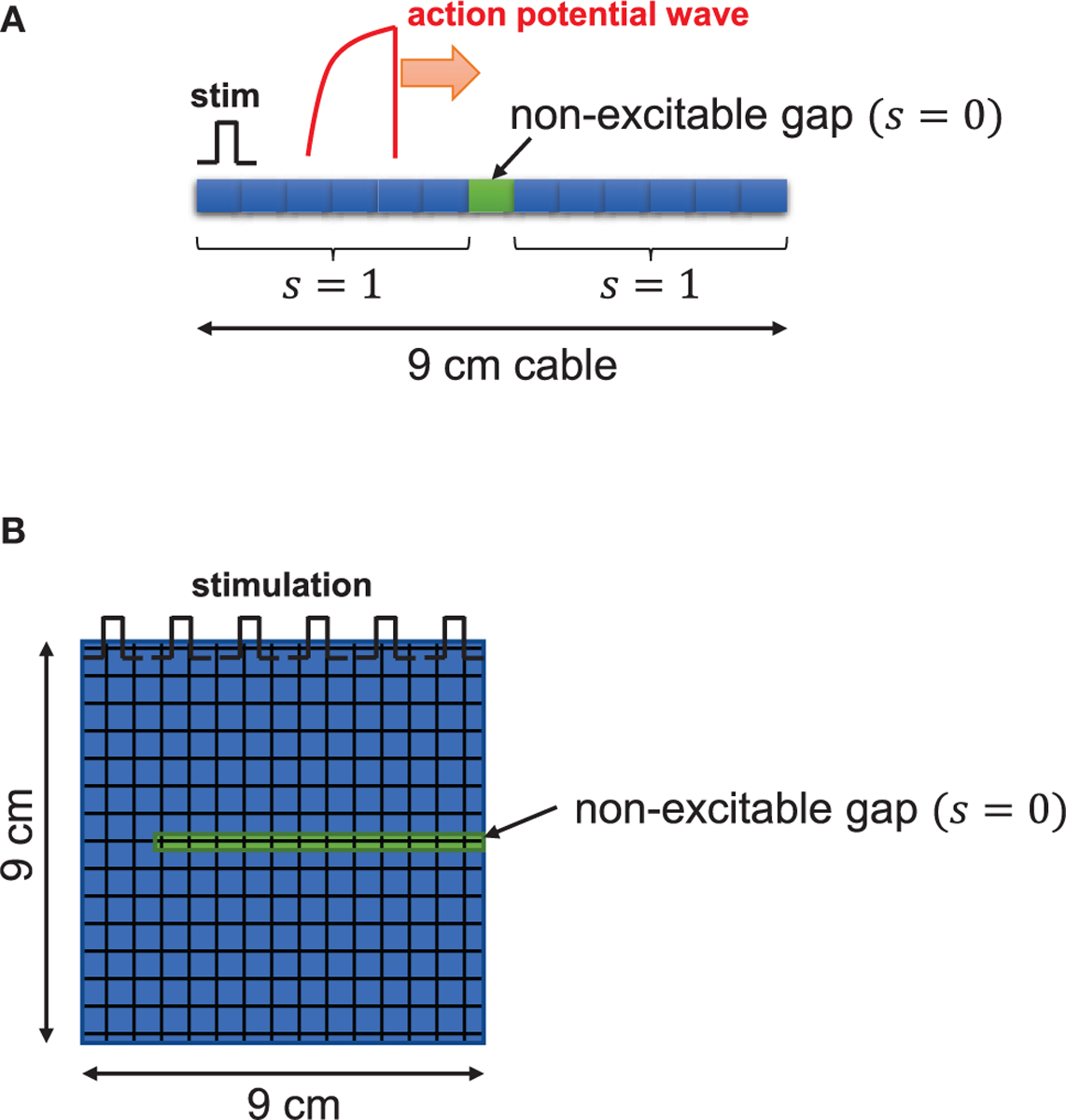
Schematic illustration. One dimensional tissue was paced with stimuli applied uniformly at the left edge of the tissue. The cable length is 9 cm. **(A)** In the cable, s(x), function of excitability with respect to position in tissue, is 1 for excitable cells and 0 for non-excitable cells. **(B)** In two-dimensional tissue, the non-excitable gap was inserted in the middle of the tissue.

We used a physiologically detailed model of the rabbit ventricular action potential model used in our previous studies [19, 36–38]. The membrane potential is governed by

$$\frac{\partial V(x,t)}{\partial t}=-s(x)\cdot \frac{I_{ion}(x,t)}{C_{m}}+\nabla \cdot D\nabla V(x,t),$$

where V is the membrane potential, $I_{ion}$ is the total transmembrane current, $C_{m}$ is the cell membrane capacitance, D is the effective diffusion constant of the voltage due to currents from neighboring cells through gap junctions, x represents position in space and t is time. In this study, we introduced the variable s(x) to control excitability in tissue as follows (Figure 1A).


$$\left\{\begin{array}{cc} s\left(x\right)=0 & \text{for}\;\text{non}\;-\;\text{excitable}\;\text{cells}\\ s\left(x\right)=1 & \text{for}\;\text{excitable}\;\text{cells} \end{array}\right.$$

Since the original parameters of this model were built based on the healthy rabbit cell data [37], EADs do not occur even at slow heart rates. In order to promote EAD formation at the cellular level, we modified parameters to reduce repolarization reserve by increasing inward current (I_CaL_) and reducing K currents. We note that even with these modifications, EADs do not occur without the non-excitable gap. EADs occur only when the nonexcitable gap is inserted in tissue and/or repolarization reserve is further reduced by increasing I_CaL_ (or decreasing K current). Parameters and equations used in this study are shown in the Supplementary Material.

### Computer Simulation

A one-dimensional cable (9 cm) was paced at one end. To ensure propagation, the leftmost five cells were paced in a one-dimensional cable. Non-excitable cells were inserted in the middle of the cable as shown in Figure 1A. The two-dimensional tissue (9 × 9 cm) was paced from the top and non-excitable cells were inserted as shown in Figure 1B. We solve this equation using the operator splitting method [39]. We use the Euler method with the variable time step of 0.01~0.1 ms to compute the single cell action potential. The space step (Δx) is 150 μm, which is similar to the length of the cardiac myocyte. For the numerical accuracy, we used double precision in our simulations and checked the results using smaller time steps. All codes are written in C/C++. We used the 25-node high-performance computing cluster.

### Simplified Model

In order to understand the dynamical mechanisms of EADs due to non-excitable gap, we also constructed the reduced mathematical model of EADs. The basic structure of the model is the same as our previous studies [40]. This model has three variables: membrane voltage (v), and gating variables (f and x). These variables are governed by

$$C_{m}\frac{dv}{dt}=-\left(i_{Ca}+i_{K}\right)+g_{gap}\left(v_{2}-v\right),$$

$$\frac{df}{dt}=\frac{f_{\infty }-f}{\tau_{f}},$$

$$\frac{dx}{dt}=\frac{x_{\infty }-x}{\tau_{x}},$$

where $i_{Ca}$ is the simplified L-type Ca^2+^ current and $i_{K}$ is the generic K current, $g_{gap}$ is the effective conductance between proximal and distal regions. $v_{2}$ is the membrane potential in the distal region. Due to the large delay at the gap region, the action potential in the distal region remains the plateau phase when the membrane potential in the proximal region is repolarizing. Thus, the membrane potential in the distal region was assumed to be constant. f and x are gating variables of the Ca^2+^ current and the generic K current, respectively. $f_{\infty }$ and $x_{\infty }$ are steady state values of f and x gates, respectively. $\tau_{f}$ and $\tau_{x}$ are time constants of f and x gates, respectively. The simplified L-type Ca^2+^ current and the generic K current are

$$i_{Ca}=g_{Ca}d_{\infty }f\left(v-e_{Ca}\right),$$

$$i_{K}=g_{k}\cdot x\cdot \left(v-e_{k}\right),$$

where $g_{Ca}$ is the maximum conductance of $i_{Ca},\;g_{k}$ is the maximum conductance of $i_{k},\;d_{\infty }$ is the instantaneous activation gate of the Ca^2+^ channel, $e_{Ca}$ is the reversal potential of $i_{Ca},\;e_{k}$ is the reversal potential of $i_{k}$. Steady state values, $d_{\infty },\;f_{\infty }$ and $x_{\infty }$ are voltage dependent and governed by

$$d_{\infty }=\frac{1}{1\;+\;exp\left(-\frac{v+32}{6.24}\right)},$$

$$f_{\infty }=\frac{1}{1\;+\;exp\left(\frac{v+21}{8.6}\right)},$$

$$x_{\infty }=\frac{1}{1\;+\;exp\left(-\frac{v+35}{5}\right)}.$$

We use the Euler method with the variable time step of 0.1 ms to solve the simplified model.

## RESULTS

**FIGURE 2 |:**
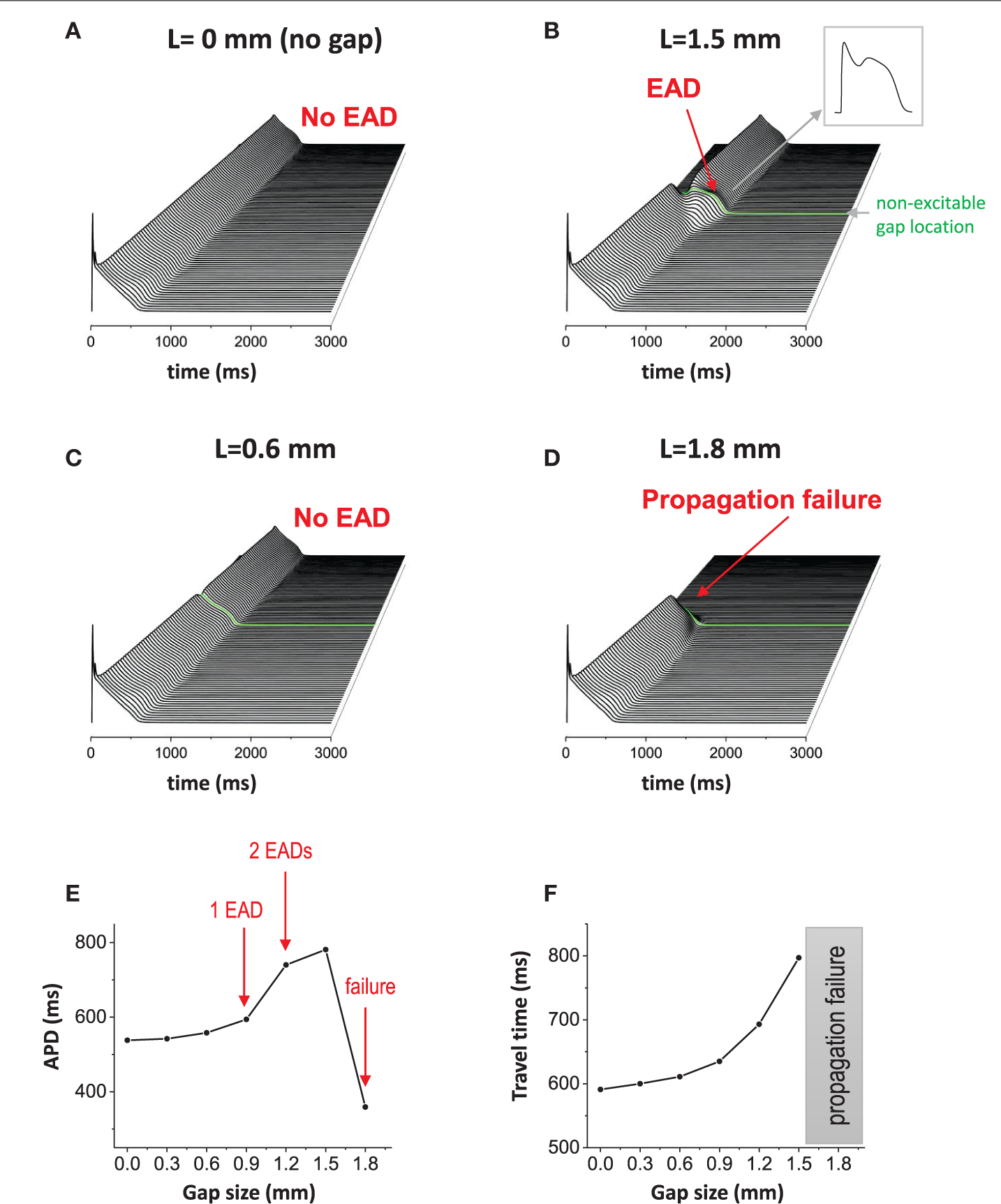
Non-excitable gap causes EADs. **(A)** Without non-excitable gap, the action potential wave propagates without causing EADs in 1D tissue. Space-time plot. **(B)** When non-excitable gap is inserted, EADs occurred around the non-excitable gap region. The maximum APD is 781 ms. The gap size is 1.5 mm. **(C)** If the gap size is too small (0.6 mm), no EADs occurred. **(D)** If the gap size is too large (1.8 mm), propagation failed at the gap region. **(E)** APD vs. the gap size. **(F)** The travel time of action potential wave vs. gap size.

One-dimensional cable was paced at one end. In this study, the cable length is 9 cm, which is longer than the typical human heart size, to avoid unnecessary boundary effects. When cells are well connected via gap junctions without a non-excitable gap, the action potential wave propagates smoothly without EADs in the cable (Figure 2A). Hereafter, we refer to this case as the “control” case. The maximum action potential duration (APD) in the cable was 538 ms in the control case. The travel time of the action potential wave from one end to the other end totaling 9 cm was 591 ms. When a non-excitable gap of 1.5 mm (= 10 cells) is inserted in the middle of the cable (Figures 1, 2B green line), the propagation speed of the action potential slows down at the gap region. In this case, the travel time of the action potential wave propagated from one end to the other end was 797 ms. Therefore, the delay of the propagation at the gap region is 259 ms. In addition, EADs occur near the gap region (Figure 2B). In this case, the maximum APD in the cable was 781 ms. The gap size is critical for the formation of EADs. If the gap size is too small, EADs do not occur (Figure 2C). EADs occurred only when the gap size reaches 1.5 mm. On the other hand, if the gap size is too large (gap size greater than or equal to 1.8 mm), the action potential wave cannot propagate due to non-excitability in the gap (Figure 2D). Figure 2E shows a graph of APD vs. gap size, and the number of EADs with different gap sizes. Additionally, the travel time of action potential wave is depicted against the gap size (Figure 2F). Note that the action potential fails to propagate if the gap size is larger than 1.8 mm.

**FIGURE 3 |:**
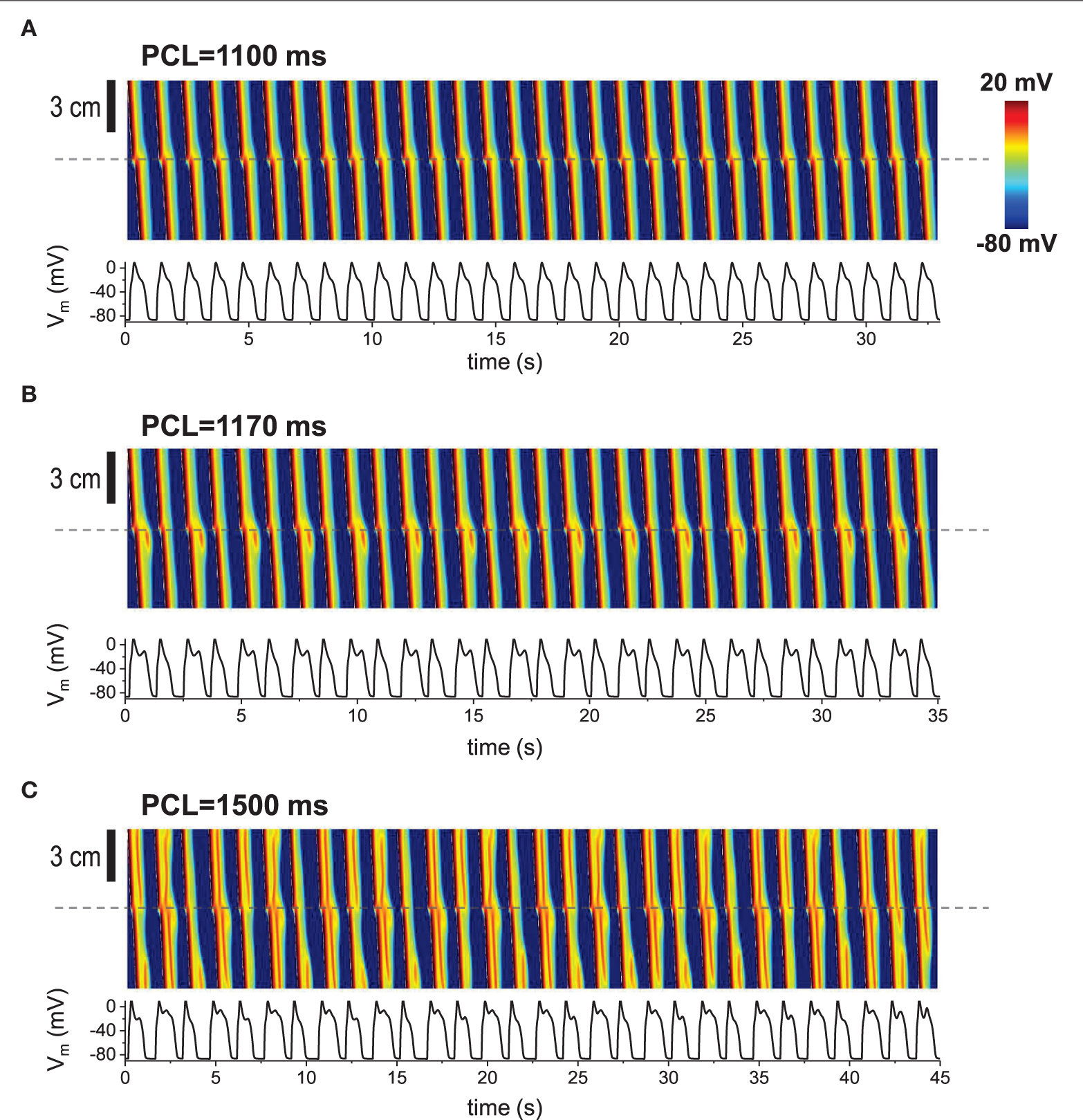
Repetitive pacing When 1D cable was paced repetitively, various patterns appeared. Top: Space-time plot. The AP traces (bottom) were taken at the middle of the cable (indicated by dashed lines). The gap size is 1.5 mm. The tissue was paced 200 times. Last 30 beats are shown here. **(A)** Periodic (period-1) pattern. No EADs at the gap region. **(B)** Period-2. EADs occur every other beat. **(C)** Complex pattern. Spatiotemporal chaos.

In our previous studies, we have shown that EADs can be periodic and chaotic due to nonlinearity of EAD dynamics [15, 19, 40–42]. When the 1D cable was paced repetitively, various patterns appeared such as periodic (period-1), period-2 and even spatiotemporally chaotic patterns (Figures 3A–C). These patterns appear only when non-excitable cells exist in tissue.

**FIGURE 4 |:**
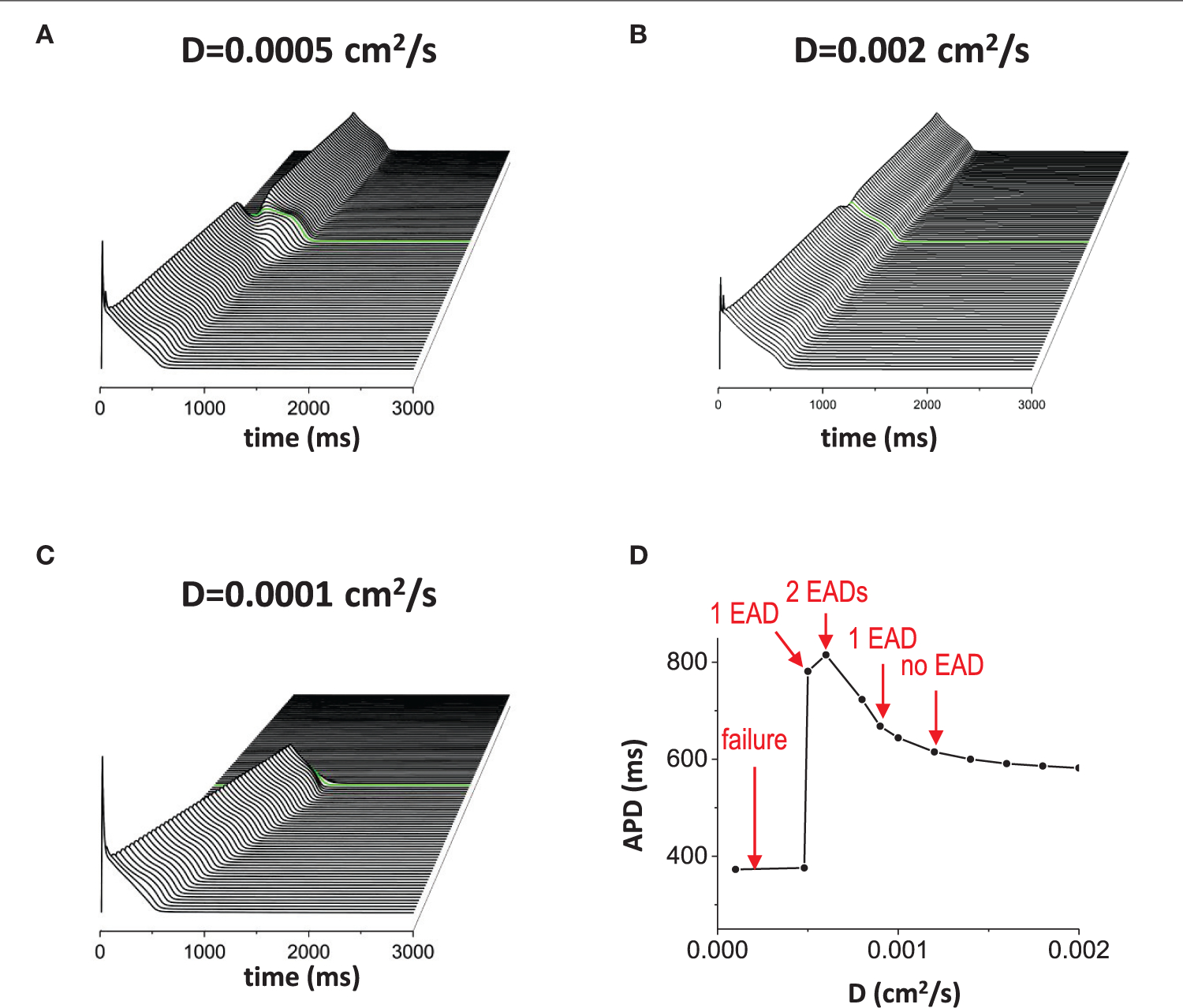
Gap junction conductance affects EAD formation. The diffusive current between cells also affects EAD formation. In this figure, the gap size was fixed at 1.5 mm. **(A)** normal D. D = 0.0005 cm^2^/s. **(B)** large D. D = 0.002 cm^2^/s. **(C)** small D. D = 0.00001 cm^2^/s. **(D)** APD vs. D.

When the non-excitable gap exists, changing the gap junction conductance also has the similar effects to the gap size since the effective gap size is proportional to 1/√D. In other words, the diffusion coefficient D rescales the length of the gap. Thus, although changing the size and changing the gap junction conductance are physiologically different, mathematically, we expect similar results. We used a fixed gap size = 1.5 mm for all simulations in Figure 4. When D is normal value ($D=0.0005{\;cm}^{2}/s$), 1 EAD was observed near the gap region (Figure 4A). If D becomes larger ($D=0.002{\;cm}^{2}/s$), the effective gap size becomes smaller and no EADs occurred (Figure 4B). If D is too small ($D=0.00001{\;cm}^{2}/s$), the action potential wave could not cross the gap region due to the large effective gap (Figure 4C). Figure 4D shows how diffusive coupling impacts EAD formation. There is an optimal window for EAD formation.

**FIGURE 5 |:**
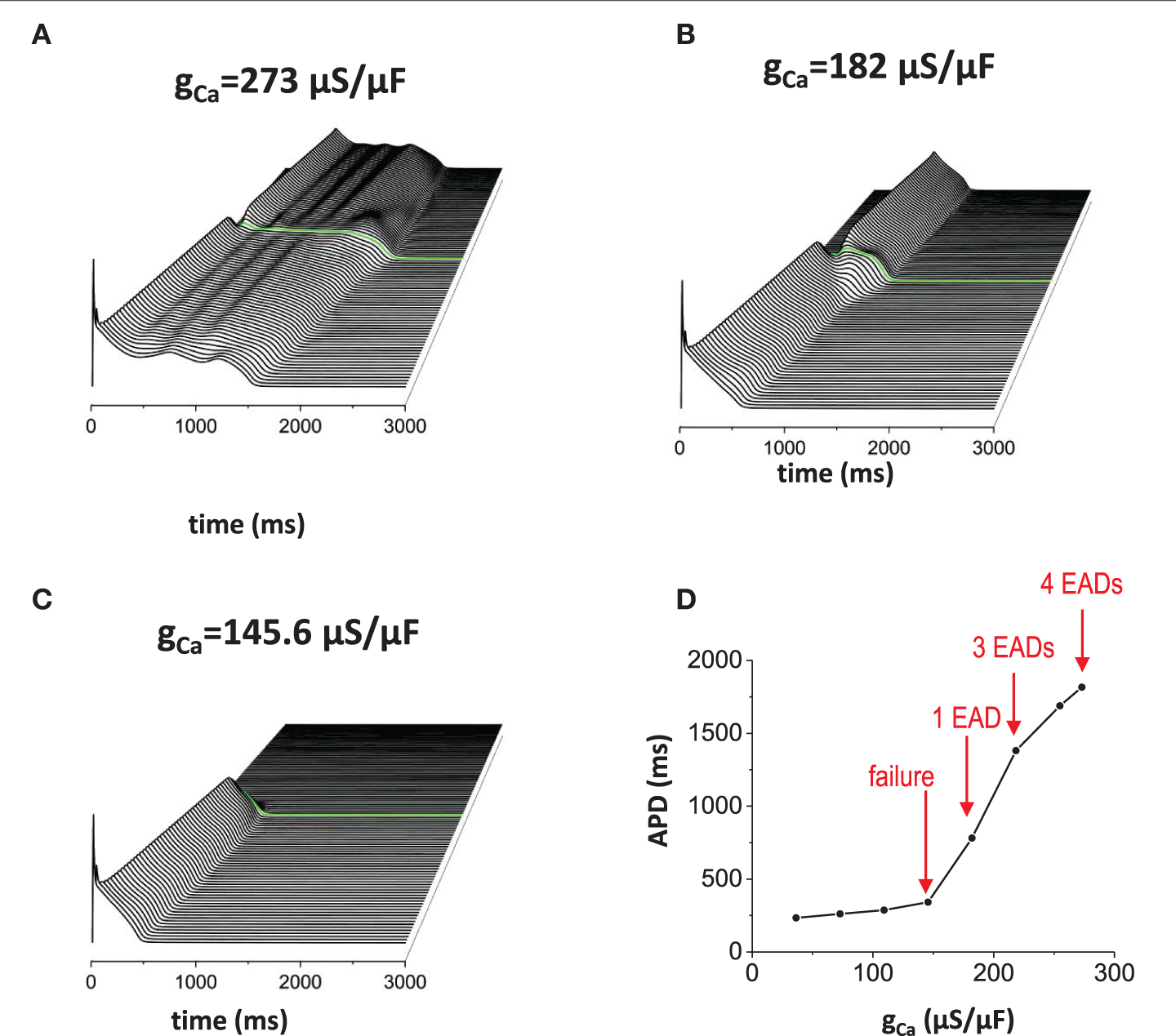
Effects of the inward current. When inward current ($I_{\mathrm{CaL}}$) is increased, EADs occur without the non-excitable gap region. In addition, non-excitable gap promotes EADs further **(A)**. $g_{Ca}=273\;\mu A/\mu F$. Without the non-excitable gap region, 3 EADs were observed. Near the gap region, 4 EADs were observed. The maximum APD is 1816 ms. **(B)** $g_{Ca}=182\;\mu A/\mu F$. Control. The maximum APD is 781 ms. **(C)** $g_{Ca}=145.6\;\mu A/\mu F$. The maximum APD is 341 ms. **(D)** a graph of APD vs. $g_{Ca}$.

Reducing repolarization reserve by increasing inward currents such as $I_{CaL}$ and/or reducing outward currents such as $I_{Kr}$ and $I_{Ks}$, promotes EAD generation. When $I_{CaL}$ is increased (Figure 5A), APD was prolonged (APD=1816ms) and the action potential wave has EADs without the non-excitable gap. When the non-excitable gap is inserted, it promoted EADs further if the gap size optimal (the gap size is 1.5 mm in Figure 5). Near the gap region, four EADs occurred whereas only three EADs occurred in the other regions. Figure 5B is the control model for comparison. If the inward current is too small, the action potential is too short to provide enough source current to initiate new action potential in the distal region even if the gap size is optimal for the control model (Figure 5C). Figure 5D is a graph of APD vs. conductance of the inward current (g_Ca_), summarizing our observation that EADs occur when the inward current increases.

**FIGURE 6 |:**
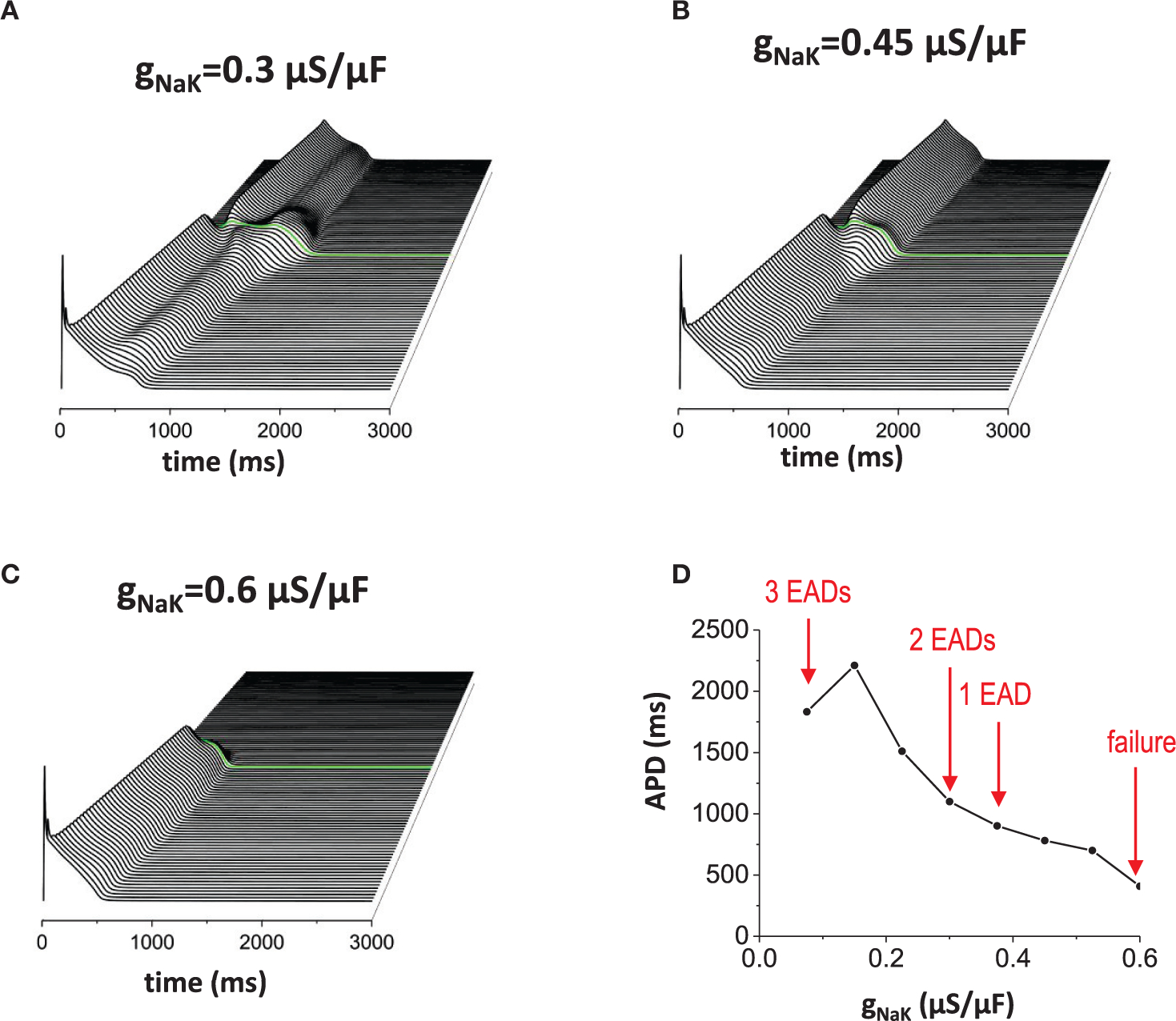
Effects of the outward current. Reducing outward current ($I_{NaK}$) also has similar effects of increasing inward current. **(A)** When the outward current was reduced ($g_{NaK}=0.3\;\mu A/\mu F$), EADs occured without the non-excitable gap region. Near the gap region, more EADs were observed (2 EADs). **(B)** $g_{NaK}=0.45\;\mu A/\mu F$. Control. **(C)** $g_{NaK}=0.6\;\mu A/\mu F$. Large outward current reduces APD. Since APD is shorter, the action potential in the proximal region cannot provide enough source current to excite cells in the distal region. **(D)** APD vs. $g_{NaK}$.

Reducing outward current also has the same effects (Figures 6A–D). When the outward current became smaller (in this case, we reduced $I_{NaK}$), more EADs occurred (Figure 6A). Figure 6B is the control model for comparison. Then, when the outward current became too large, the action potential wave failed to propagate (Figure 6C). To summarize these results, we plotted a graph of APD against conductance of the outward current (g_NaK_; Figure 6D).

To understand the dynamical mechanisms of EADs due to the non-excitable gap, we analyzed using a simplified mathematical model of EADs (see Material and Methods). In this model, v is the membrane potential of the cell in the proximal region near the gap. We assessed how current from the distal region promotes EAD generation. We assume the membrane potential of the cell in the distal region ($v_{2}$) remains in the plateau phase due to the large delay (>200 ms) of the propagation at the gap region. Since x is the slowest variable in this system, we take it as a parameter. Then, the 2-variable system can be written as

$$\frac{dv}{dt}=F(v,f)=\;-\left(g_{Ca}d_{\infty }f\left(v-e_{Ca}\right)+g_{k}\cdot x\cdot \left(v-e_{k}\right)\right)\;\;+g_{gap}\left(v_{2}-v\right),\;\frac{df}{dt}=G(v,f)=\frac{f_{\infty }-f}{\tau_{f}},$$

where

$$d_{\infty }=\frac{1}{1\;+\;exp\left(-\frac{v+32}{6.24}\right)},$$

$$f_{\infty }=\frac{1}{1\;+\;exp\left(\frac{v+21}{8.6}\right)}.$$

Thus, the matrix to compute the stability of the system is

$$M\;=\left(\begin{array}{ll} F_{v} & F_{f}\\ G_{v} & G_{f} \end{array}\right)\;\;=\left(\begin{array}{cc} -g_{Ca}d_{\infty }^{\prime }fv-g_{Ca}d_{\infty }f-g_{k}x+g_{gap} & -g_{Ca}d_{\infty }v\\ f_{\infty }^{\prime }/\tau_{f} & -1/\tau_{f} \end{array}\right)$$

**FIGURE 7 |:**
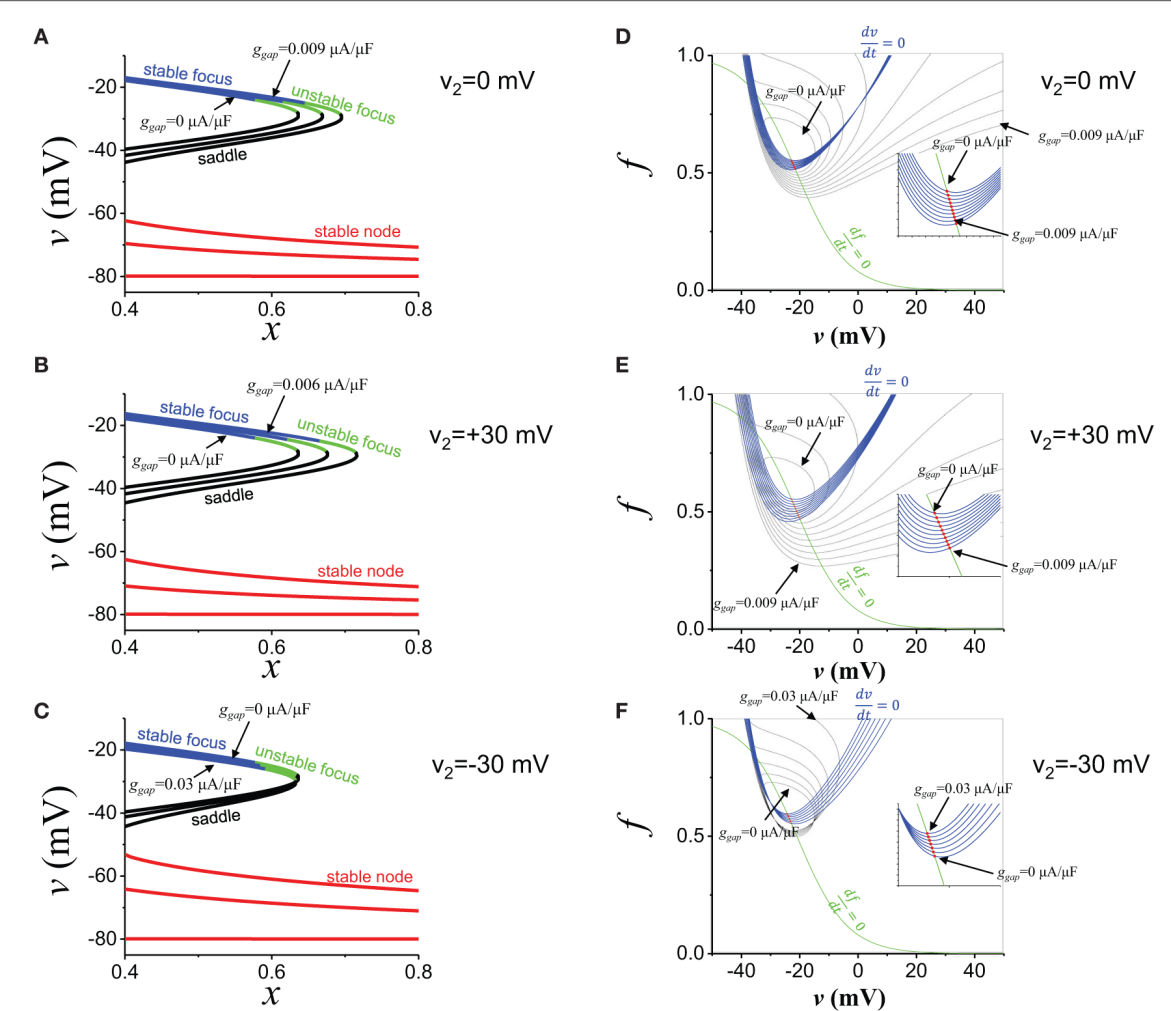
EAD mechanisms. **(A)** The stability of the fats subsystem (v,f). x is the parameter. Blue: stable focus. Green: unstable focus. Black: saddle. Red: stable node. $g_{gap}$ values are 0, 0.0045, and 0.009 μA/μF. $v_{2}$ is 0 mV. **(B)** The same as **(A)**, but $v_{2}$ is +30 mV. $g_{gap}$ values are 0, 0.003, and 0.006 μA/μF. **(C)** The same as **(A)**, but $v_{2}$ is−30 mV. $g_{gap}$ values are 0, 0.015, and 0.03 μA/μF. **(D)** Basin of attraction. $g_{gap}$ value was varied from 0 to 0.009 μA/μF. $v_{2}$ is 0 mV. Red dots indicate fixed points. Blue and green lines are nuclines. **(E)** The same as **(D)**, but $v_{2}$ is +30 mV. $g_{gap}$ value was varied from 0 to 0.009 μA/μF. **(F)** The same as **(D)**, but $v_{2}$ is −30 mV. $g_{gap}$ value was varied from 0 to 0.03 μA/μF.

Figures 7A–C show the effects of the current from the distal region. As the gap junction conductance is increased (from $g_{\mathrm{gap}}=0$ to $g_{\mathrm{gap}}=0.009\;\mu A/\mu F$), the attractor region (blue part in Figure 7A) was extended and repeller (green part in Figure 7A) became attractor (unstable focus → stable focus). We varied the distal membrane potential from +30 mV to −30 mV (Figures 7B,C). In all cases, the current from the distal region promoted oscillatory attractors. We note that if the distal membrane potential becomes lower than ~−30 mV, the current from the distal region suppresses EADs. We also computed basins of attraction (Figures 7D–F). In all cases, the basin of attraction was increased as the gap junction conductance was increased. However, as the distal membrane potential becomes lower, larger conductance was required to extend the basin of attraction (Figure 7E vs. Figure 7F). On the other hand, the current from the distal region has little effect on fix points (red dots in Figure 7) and EAD oscillations always occur near −20 mV.

**FIGURE 8 |:**
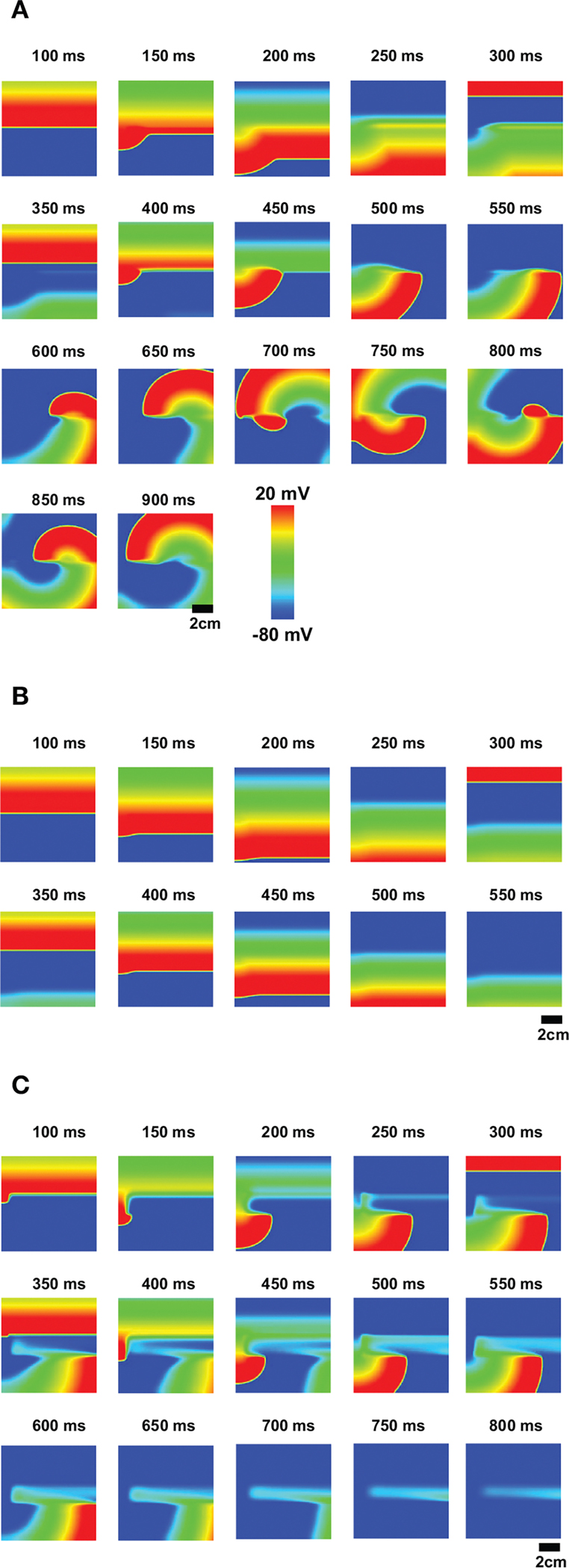
EADs due to the non-excitable gap region cause reentry. **(A)** The optimal non-excitable gap region (1.2 mm) cause EADs. These EADs block the next action potential wave and cause reentry. The tissue size is 9 × 9 cm. (Supplemental Movie 1; **B)** If the gap size is too small, EADs will not occur although there is a delay at the gap region. (Supplemental Movie 2; **C)** If the gap size is too large, the action potential wave cannot propagate and the wave avoids this region (Supplemental Movie 3).

When non-excitable cells are inserted in 2-dimensional tissue, EADs caused by the non-excitable gap region can initiate reentry, which has been associated with ventricular tachycardia. Without non-excitable gap, the action potential wave propagates smoothly. When non-excitable gap is inserted (Figure 1C), EADs occur near this region. EADs prolong APD. Thus, if cells in this region cannot recover by the time the next wave arrives, the wave cannot propagate in this region. This large dispersion of refractoriness and regional block of the wave can cause reentry (Figure 8A, Supplemental Movie 1). If the gap size is too small to cause EADs, although there is a small delay of the propagation at the gap region, dispersion of refractoriness is much smaller and rarely causes reentry (Figure 8B, Supplemental Movie 2). If the gap size is too large, any waves cannot propagate in this region and waves go around this region (Figure 8C , Supplemental Movie 3). These results demonstrate that non-excitability in tissue can lead to large dispersion of refractoriness and may cause arrhythmias.

## DISCUSSION

Tissue heterogeneity has been thought to be one of the contributing factors of arrhythmias [43–47]. In this study, we investigated how non-excitable cells in excitable tissue promote EADs and thus arrhythmias.

EADs can be caused by reactivation of the Ca^2+^ channels, spontaneous Ca^2+^ releases from the SR, or both [10–15]. In addition, recent studies have shown that reactivation of the Na^+^ channels can also lead to EADs [15, 48]. In this study, we showed the mechanism of Ca^2+^ channel-mediated EADs due to heterogeneously distributed excitable and non-excitable cells, and demonstrated reentrant arrhythmias in 2D tissue using physiologically detailed computational models. It has been well-studied how non-excitable cells in excitable tissue can lead to reflected waves [23–29]. In these studies, the key to the reflected waves was reactivation of the Na^+^ channels. In this study, we showed the reflection occurs at the plateau voltage due to reactivation of the Ca^2+^ channels. In our simulations, EADs did not cause a retrograde wave since the amplitude of EADs was too small but prolonged APDs.

The number of EADs is sensitive to the gap size (Figure 2). The detailed analysis using a two-variable model has shown that there are infinite patterns of reflected waves (1 reflected wave, 2 reflected waves, 3 reflected waves . . . infinite reflected waves) between normal propagation (no reflected waves) and propagation failure when the gap size is varied [33]. In our study, we did not observe these patterns even when the gap size is finely tuned. This is probably because the memory effect in the model interfered the patterns.

In tissue, large dispersion of refractoriness can initiate reentry [49]. When non-excitable cells are inserted in 2D tissue, dispersion of refractoriness can be observed without EADs (Figure 8B, Supplemental Movie 2). However, the dispersion is small in this case. When EADs occur, the dispersion becomes large enough to initiate reentry (Figure 8A, Supplemental Movie 1). The non-excitable gap also becomes an anchor of the spiral waves (Supplemental Movie 1). But no EADs were observed since the cycle of the rotation of the spiral wave was too fast for EADs. If the gap size is too large, the gap region blocks action potential waves (Figure 8C, Supplemental Movie 3). It is known that obstacles in tissue can lead to reentry [50, 51]. Thus, the large gap region can also initiate reentry by different mechanisms.

It is known that tissue geometry is also an important factor for reflected waves [32]. When an action potential wave propagates from a narrow path to a wide path, the propagation speed slows down due to the source-sink mismatch. This delay can re-excite the cells in the narrow path and cause a retrograde wave. Thus, propagation delay due to tissue geometry may be able to initiate EADs. This possibility needs to be investigated.

Reflected waves have been observed and investigated in various systems such as cardiac tissue and neurons. The cardiac subcellular Ca^2+^ system is also an excitable system. Under normal conditions, Ca^2+^ release from the sarcoplasmic reticulum (SR) forms a spark. However, when Ca^2+^ sparks recruit new Ca^2+^ sparks in neighboring Ca^2+^ release units, Ca^2+^ sparks propagate as a wave [52, 53]. Each Ca^2+^ release unit contains a few to several hundred ryanodine receptors [54–58] and the number of ryanodine receptors affects the positive feedback process known as Ca^2+^-induced Ca^2+^ release [58, 59]. In addition, the subcellular structure is very complex. These subcellular heterogeneities may lead to reflection and form complex patterns in Ca^2+^ waves.

In this paper, we showed only mathematical and computational results. These results should be verified in experiments. Reflected waves have been observed in many wet experiments. We expect that we can use the same experimental setup for EADs due to non-excitable cells. However, to observe EADs, repolarization reserve needs to be reduced.

## CONCLUSIONS

Cellular mechanisms of EADs have been widely studied. In this study, we showed that tissue properties are also critical for initiation and promotion of EADs. Non-excitable gap in tissue can promote EADs and prolonged action potentials due to EADs can cause conduction block and reentry of the action potential wave.

The limitation of this study is that we considered only Ca^2+^ channel reactivation. Retrograde waves propagate when the Na^+^ channels are reactivated. If both cases are considered, focal and reentrant arrhythmias can coexist and the dynamics will become much more complex.

Ablation creates non-excitable tissue. The border zone of myocardial infarction is also mixture of excitable and non-excitable cells. Our study implies that EADs can be promoted in these regions due to tissue heterogeneity. This study sheds light on the role of tissue heterogeneity on EAD generation and initiation of reentrant arrhythmias.

## Supplementary Material

Data Sheet 1.pdf

Video 2.MP4

Video 3.MP4

Video 1.MP4

The Supplementary Material for this article can be found online at: https://www.frontiersin.org/articles/10.3389/fphy.2018.00117/full#supplementary-material

Supplemental Movie 1 | Movie of Figure 8A.

Supplemental Movie 2 | Movie of Figure 8B.

Supplemental Movie 3 | Movie of Figure 8C.
