# Supplementary material for: Mathematical Analysis of the Role of Heterogeneous Distribution of Excitable and Non-excitable Cells on Early Afterdepolarizations
Source: Front Phys. Author manuscript; Available in PMC 2025 Sep 4. (PMC12407153; doi:10.3389/fphy.2018.00117)
Supplement: Data Sheet 1.pdf [file NIHMS2105073-supplement-Data_Sheet_1_pdf.pdf]

frontiers

Supplementary Material

Mathematical Analysis of the Role of Heterogeneous Distribution of Excitable and Non-excitable Cells on Early Afterdepolarizations

Seunghyun Kim and Daisuke Sato*

*** Correspondence:** Daisuke Sato (dsato@ucdavis.edu)

1 Cell model

In this study, we used the physiologically detailed rabbit ventricular action potential developed by Mahajan *et al*.(1). This model is based on the rabbit ventricular action potential model by Shannon *et al.*, (2) and intracellular Ca cycling model by Shiferaw et al (3).

1.1 Membrane voltage dynamics

The membrane voltage (V) is described by

$$C_m \frac{dV}{dt} = -(I_{ion} + I_{stim}),$$

where C_m=1 μF/cm^2 is the membrane capacitance, I_{ion} is the total ionic current density, and I_{stim} is the stimulus current (typically 80 μF/μA and duration 2 ms). In this model, the total ionic current is

$$I_{ion} = I_{Na} + I_{to,f} + I_{to,s} + I_{Kr} + I_{Ks} + I_{K1} + I_{NaK} + I_{Ca} + I_{NaCa}.$$

Parameter values of the currents were kept the same as in Shannon *et al.* (2), and are listed in the tables.

The model was integrated using the Euler method with an adaptive time step ranging from 0.1 to 0.01 ms.

1.2 Ionic Currents

1.2.1 The fast sodium current (I_{Na})

Original formulation due to Luo and Rudy (4) and used in the Shannon model :

$$
\begin{aligned}
I_{Na} &= g_{Na} m^3 h j (V - E_{Na}),\\
E_{Na} &= \frac{RT}{F} \ln\left(\frac{[Na^+]_o}{[Na^+]_i}\right),\\
\frac{dh}{dt} &= \alpha_h (1-h) - \beta_h h,\\
\frac{dj}{dt} &= \alpha_j (1-j) - \beta_j j,\\
\frac{dm}{dt} &= \alpha_m (1-m) - \beta_m m,\\
\alpha_m &= 0.32 \frac{V + 47.13}{1 - e^{-0.1(V+47.13)}},\\
\beta_m &= 0.08 e^{-\frac{V}{11}}.
\end{aligned}
$$

For $V \geq -40$ mV

$$
\begin{aligned}
\alpha_h &= 0,\\
\alpha_j &= 0,\\
\beta_h &= \frac{1}{0.13\left(1 + e^{\frac{V+10.66}{-11.1}}\right)},\\
\beta_j &= 0.3 \frac{e^{-2.535\times 10^{-7} V}}{1 + e^{-0.1(V+32)}}.
\end{aligned}
$$

For $V < -40$ mV

$$
\begin{aligned}
\alpha_h &= 0.135 e^{\frac{V+80}{-6.8}},\\
\beta_h &= 3.56 e^{0.079V} + 3.1 \times 10^5 e^{0.35V},\\
\alpha_j &= \frac{[(-1.2714 \times 10^5 e^{0.2444V} - 3.474 \times 10^{-5} e^{-0.04391V})(V + 37.78)]}{[1 + e^{0.311(V+79.23)}]},\\
\beta_j &= \frac{0.1212 e^{-0.01052V}}{1 + e^{-0.1378(V+40.14)}}.
\end{aligned}
$$

1.2.2 Inward rectifier K+ current (I_{K1})

Formulation due to Luo and Rudy and used in the Shannon model.

$$
\begin{aligned}
I_{K1} &= g_{K1}\sqrt{\frac{[K^+]_o}{5.4}}\frac{A_{K1}}{A_{K1}+B_{K1}}(V-E_K),\\
A_{K1} &= \frac{1.02}{1.0+e^{0.2385(V-E_K-59.215)}},\\
B_{K1} &= \frac{0.49124e^{0.08032(V-E_K+5.476)}+e^{0.061750(V-E_K-594.31))}}{1+e^{-0.5143(V-E_K+4.753)}},\\
E_K &= \frac{RT}{F}\ln\left(\frac{[K^+]_o}{[K^+]_i}\right).
\end{aligned}
$$

1.2.3 The Rapid component of the delayed rectifier K+ current (I_{Kr})

Formulation due to Luo and Rudy, with modifications for the rabbit myocyte due to Puglisi *et al*. (5), and as implemented in the Shannon model.

$$
\begin{aligned}
I_{Kr} &= g_{Kr}\sqrt{\frac{[K^+]_o}{5.4}}x_{Kr}R(V)(V-E_K),\\
R(V) &= \frac{1}{1+e^{\frac{V+33}{22.4}}},\\
\frac{dx_{Kr}}{dt} &= \frac{x_{Kr}^{\infty}-x_{Kr}}{\tau_{Kr}},\\
x_{Kr}^{\infty} &= \frac{1}{1+e^{-\frac{(V+50)}{7.5}}},\\
\tau_{Kr} &= \frac{1}{\left(\frac{0.00138(V+7)}{1-e^{-0.123(V+7)}}+\frac{0.00061(V+10)}{-1+e^{0.145(V+10)}}\right)}
\end{aligned}
$$

1.2.4 The slow component of the delayed rectifier K current (I_{Ks})

Originally formulated by Luo and Rudy and implemented as in the Shannon model. The Ca dependence of the conductance was modified to fit dynamic restitution data.

$$I_{Ks} = g_{Ks} x_{ks}^2 q_{Ks} (V - E_{Ks}),$$
$$\frac{dx_{Ks}}{dt} = \frac{x_{Ks}^{\infty} - x_{Ks}}{\tau_{Ks}},$$
$$x_{Ks}^{\infty} = \frac{1}{1 + e^{-(V-1.5)/16.7}},$$
$$\tau_{Ks} = \frac{1}{\left(\frac{0.0000719(V+30)}{1 - e^{-0.148(V+30)}} + \frac{0.000131(V+30)}{-1 + e^{0.0687(V+30)}}\right)},$$
$$E_{Ks} = \frac{RT}{F} \ln\left(\frac{[K^+]_o + 0.01833[Na^+]_o}{[K^+]_i + 0.01833[Na^+]_i}\right)$$

The slow Ca dependent increase in the conductance is modelled by treating q_{Ks} as a dynamical variable that obeys the equation

$$\frac{dq_{Ks}}{dt} = \frac{q_{Ks}^{\infty} - q_{Ks}}{\tau_{qKs}},$$

where $q_{Ks}^{\infty} = 0.6c_s$, and where τ_{qKs}=1000 ms.

1.2.5 The NaK exchanger current (I_{NaK})

As in the Shannon model.

$$\sigma = \frac{e^{\frac{[Na^+]_o}{67.3}} - 1}{7},$$
$$f_{NaK} = \frac{1}{1 + 0.1245 e^{-\frac{0.1VF}{RT}} + 0.0365\sigma e^{-\frac{VF}{RT}}},$$
$$I_{NaK} = g_{NaK} f_{NaK} \left(\frac{1}{1 + \left(\frac{12}{[Na^+]_i}\right)}\right)\left(\frac{[K^+]_o}{[K^+]_o + 1.5}\right).$$

1.2.6 The fast component of the rapid inward K+ current ($I_{to,f}$)

As in the Shannon model.

$$I_{to,f} = g_{to,f} X_{to,f} Y_{to,f} (V - E_K),$$
$$X_{to,f}^{\infty} = \frac{1}{1 + e^{-\frac{(V+3)}{15}}},$$
$$Y_{to,f}^{\infty} = \frac{1}{1 + e^{\frac{(V+33.5)}{10}}},$$
$$\tau_{Xto,f} = 3.5 e^{-\left(\frac{V}{30}\right)^2} + 1.5,$$
$$\tau_{Yto,f} = \frac{20}{1 + e^{\frac{V+33.5}{10}}} + 20,$$
$$\frac{dX_{to,f}}{dt} = \frac{X_{to,f}^{\infty} - X_{to,f}}{\tau_{Xto,f}},$$
$$\frac{dY_{to,f}}{dt} = \frac{Y_{to,f}^{\infty} - Y_{to,f}}{\tau_{Yto,f}}.$$

1.2.7 The slow component of the rapid inward K+ current ($I_{to,s}$)

$I_{to,s}$ is given by

$$I_{to,s} = g_{to,s} X_{to,s} \left(Y_{to,s} + 0.5 R_s^{\infty}\right)(V - E_K),$$
$$R_s^{\infty} = \frac{1}{1 + e^{\frac{V+33.5}{10}}},$$
$$X_{to,s}^{\infty} = \frac{1}{1 + e^{-\frac{V+3}{15}}},$$
$$Y_{to,s}^{\infty} = \frac{1}{1 + e^{\frac{V+33.5}{10}}},$$
$$\tau_{Xto,s} = \frac{9}{1 + e^{\frac{V+3}{15}}} + 0.5,$$
$$\tau_{Yto,s} = \frac{3000}{1 + e^{\frac{V+60}{10}}} + 30,$$
$$\frac{dX_{to,s}}{dt} = \frac{X_{to,s}^{\infty} - X_{to,s}}{\tau_{Xto,f}},$$
$$\frac{dY_{to,s}}{dt} = \frac{Y_{to,s}^{\infty} - Y_{to,s}}{\tau_{Yto,s}}.$$

1.3 Intracellular calcium cycling

Intracellular Ca cycling in the rabbit myocyte is described using a model due to Shiferaw *et al*. (3). The equations for Ca cycling are:

$$\frac{dc_s}{dt} = \beta_s \left[\frac{v_i}{v_s}(J_{rel} - J_d + J_{Ca} + J_{NaCa}) - J_{trpn}^s\right],$$
$$\frac{dc_i}{dt} = \beta_i\left[J_d - J_{up} + J_{leak} - J_{trpn}^i\right],$$
$$\frac{dc_j}{dt} = -J_{rel} + J_{up} - J_{leak},$$
$$\frac{dc_j'}{dt} = \frac{c_j - c_j'}{\tau_a},$$
$$\frac{dJ_{rel}}{dt} = N_s'(t) \cdot Q(c_j') - \frac{J_{rel}}{\tau_r}$$

where c_s, c_i, and c_j are the average concentrations of free Ca in a thin layer just below the cell membrane (the submembrane space), in the cytosol, and the SR, with volumes v_s, v_i, and v_{sr}, respectively. Here, the SR volume includes both the junctional SR (JSR) and the network SR (NSR). Also c_j' is the average JSR concentration within dyadic junctions in the whole cell. The concentrations c_s and c_i are in units of μM, whereas c_j and c_j' are in units of μM v_{sr}/v_i (μM/l cytosol).

The current fluxes are: J_{rel}, the total release flux out of the SR via RyR channels; J_d, the diffusion of Ca from the submembrance space to the bulk myoplasm; J_{up}, the uptake current via SERCA channels on the SR; J_{Ca}, the current flux into the cell via L-type Ca channels; J_{NaCa}, the current flux into the cell via the NaCa exchanger; J_{leak}, the leak current from the SR into the bulk myoplasm.

All Ca fluxes are divided by v_i and have units of μM/ms, which can be converted to units of μA/ μF using the conversion factor nFv_i/C_m, where n is the ionic charge of the current carrier, C_m is the cell membrane capacitance, and where F is Faraday's constant. Thus, ionic fluxes can be converted to membrane currents using

$$I_{Ca} = -2\alpha J_{Ca},$$
$$I_{NaCa} = \alpha J_{NaCa},$$

where $\alpha = Fv_i/C_m$, and where the ion currents are in units of μA/μF.

1.3.1 The L-type Ca current

The Ca flux into the cell due to the L-type Ca current is given by

$$J_{ca} = g_{Ca} P_o i_{Ca},$$
$$i_{Ca} = \frac{4VF^2}{RT} \frac{\tilde{c}_s e^{2a} - 0.341[Ca^{2+}]_o}{e^{2a} - 1},$$

where $a=VF/RT$, and where $\tilde{c}_s$ is the submembrane concentration in units of mM.

The open probability of the L-type Ca current is dictated by the kinetics of the Markov model shown in Mahajan *et al*. (1). The equations for the Markov states are

$$\frac{dC_2}{dt} = \beta C_1 + k_5 I_{2Ca} + k_5' I_{2Ba} - (k_6 + k_6' + \alpha) C_2,$$
$$\frac{dC_1}{dt} = \alpha C_2 + k_2 I_{1Ca} + k_2' I_{1Ba} + r_2 P_o - (r_1 + \beta + k_1 + k_1') C_1,$$
$$\frac{dI_{1Ca}}{dt} = k_1 C_1 + k_4 I_{2Ca} + s_1 P_o - (k_2 + k_3 + s_2) I_{1Ca},$$
$$\frac{dI_{2Ca}}{dt} = k_3 I_{1Ca} + k_6 C_2 - (k_4 + k_5) I_{2Ca},$$
$$\frac{dI_{1Ba}}{dt} = k_1' C_1 + k_4' I_{2Ba} + s_1' P_o - (k_2' + k_3' + s_2') B_{1Ba},$$
$$\frac{dI_{2Ba}}{dt} = k_3' I_{1Ba} + k_6' C_2 - (k_5' + k_4') I_{2Ba},$$

where the open probability satisfies

$$P_o = 1 - (C_1 + C_2 + I_{1Ca} + I_{2Ca} + I_{1Ba} + I_{2Ba}).$$

The rates are given by:

$$\alpha = p_o^\infty / \tau_{po}$$
$$\beta = \frac{1 - p_o^\infty}{\tau_{po}}$$
$$p_o^\infty = \frac{1}{1 + e^{-V/8}},$$
$$s_1 = 0.0182688 f(c_p),$$

$$
\begin{aligned}
k_1 &= 0.024168 f(c_p),\\
s_2 &= s_1(k_2/k_1)(r_1/r_2),\\
s_2' &= s_1'(k_2'/k_1')\,(r_1/r_2),\\
f(c_p) &= \frac{1}{1+\left(\widetilde{c_p}/c_p\right)^3},\\
k_3 &= \frac{e^{-\frac{(V+40)}{3}}}{3(1+e^{-(V+40)/3})},\\
k_3' &= k_3,\\
k_4 &= k_3(\alpha/\beta)(k_1/k_2)(k_5/k_6),\\
k_4' &= k_3'(\alpha/\beta)(k_1'/k_2')(k_5'/k_6'),\\
k_5 &= \frac{1-P_s}{\tau_{Ca}},\\
k_6 &= \frac{f(c_p)P_s}{\tau_{Ca}},\\
k_5' &= \frac{1-P_s}{\tau_{Ba}},\\
k_6' &= \frac{P_s}{\tau_{Ba}},\\
\tau_{Ca} &= (R(V)-T_{Ca})P_r + T_{Ca},\\
\tau_{Ba} &= (R(V)-T_{Ba})P_r + T_{Ba},\\
T_{Ca} &= \frac{78.0329}{1+\left(\frac{c_p}{\bar{c}_p}\right)^4},\\
R(V) &= 10 + 4954 e^{\frac{V}{15.6}},\\
P_r &= \frac{e^{-\frac{(V+40)}{4}}}{1+e^{-\frac{(V+40)}{4}}},\\
P_s &= \frac{1}{1+e^{-\frac{(V+40)}{11.32}}}.
\end{aligned}
$$

The dependence of Ca release on SR Ca load is determined by the function $Q(c_j')$, which has the functional form

$$
Q(c_j') = \begin{cases} 0 & 0 < c_j' < 50 \\ c_j' - 50 & 50 \le c_j' \le c_{sr}, \\ uc_j' + s & c_j' > c_{sr} \end{cases}
$$

which is the local Ca release at a dyadic junction in units of 10^{-6}μM/ms. The slope u controls the slope of the SR Ca release vs. SR Ca load relationship at high loads i.e. for $c_j > c_{sr}$.

The formulation of Ca release from the SR is modelled by computing the rate at which sparks are recruited in the whole cell. The rate of spark recruitment N_s' is proportional to the total Ca entry into the cell

$$N_s' = g_{RyR}(V) P_o i_{Ca},$$

where $g(V)$ is the gain function, which controls the voltage dependence of Ca released into the SR in response to a trigger from the L-type Ca current. This quantity has units of sparks/ms. The voltage dependence has the form

$$g_{RyR}(V) = g_{RyR} \frac{e^{-b(V+30)}}{1 + e^{-b(V+30)}},$$

where the parameter b is chosen so that the voltage dependence of release is shifted roughly by 10 mV with respect with the whole cell Ca entry.

Ca induced inactivation was modelled phenomenologically. c_p is the average concentration of Ca in the vicinity of L-type Ca channels and have the form

$$\frac{dc_p}{dt} = \tilde{J}_{SR} + \tilde{J}_{Ca} - \frac{c_p - c_s}{\tau_d},$$

$$\tilde{J}_{Ca} = -\tilde{g}_{Ca} P_o i_{Ca},$$

$$\tilde{J}_{SR} = G_{SR}(V) P_o Q(c_{jsr}),$$

$$G_{SR}(V) = \tilde{g}_{SR} \frac{e^{-b(V+30)}}{1 + e^{-b(V+30)}},$$

where $\tilde{J}_{SR}$ and $\tilde{J}_{Ca}$ are the components due to the Ca fluxes from the SR and L-type Ca current respectively, while the last term denotes the diffusion of Ca into the submembrane space.

Then, Ca induced inactivation is modeled by letting the inactivation rate constants obey

$$s_1 = s_1^0 f(c_p),$$

$$k_1 = k_1^0 f(c_p),$$

where

$$f(c_p) = \frac{1}{1 + (\tilde{c}_p / c_p)^3}$$

1.3.2 Diffusive flux

Following Shiferaw *et al.* (3) the flux of Ca from the submembrane space to the bulk myoplasm is described by

$$J_d = \frac{c_s - c_i}{\tau_d}.$$

1.3.3 Nonlinear Buffering

Instantaneous buffering of Ca to SR and calmodulin sites in the submembrane and bulk myoplasm are modelled following Shannon et al. (6), and are described by

$$\beta_s = \left(1 + \frac{B_{SR} + K_{SR}}{(c_s + K_{SR})^2} + \frac{B_{cd} + K_{cd}}{(c_s + K_{cd})^2}\right)^{-1}$$
$$\beta_i = \left(1 + \frac{B_{SR} + K_{SR}}{(c_i + K_{SR})^2} + \frac{B_{cd} + K_{cd}}{(c_i + K_{cd})^2}\right)^{-1}.$$

Time dependent buffering to Troponin C is described by

$$\frac{d[CaT]_i}{dt} = J_{trpn}^i,$$
$$\frac{d[CaT]_s}{dt} = J_{trpn}^s,$$
$$J_{trpn}^i = k_{on}^T c_i (B_T - [CaT]_i) - k_{off}^T [CaT]_i,$$
$$J_{trpn}^s = k_{on}^T c_s (B_T - [CaT]_s) - k_{off}^T [CaT]_s,$$

1.3.4 The NaCa exchange (NCX) flux

The flux of Ca due to the NaCa exchanger is based on the formulation of Luo and Rudy (4), with modifications in ref (7). The equation is

$$J_{NaCa} = g_{naca} \cdot K_a \cdot \frac{e^{\xi a}[Na^+]_i^3[Ca^{2+}]_o - e^{(\xi-1)a}[Na^+]_o^3 c_s}{(1 + k_{sat}e^{(\xi-1)a}) \cdot H},$$

where

$$H = K_{m,Cao}[Na^+]_i^3 + K_{m,Nao}^3 \cdot c_s + K_{m,Nai}^3[Ca^{2+}]_o \cdot \left(1 + \frac{c_s}{K_{m,Cai}}\right) + K_{m,Cai}[Na^+]_o^3 \cdot \left(1 + \frac{[Na^+]_i^3}{K_{m,Nai}^3}\right) + [Na^+]_i^3 \cdot [Ca^{2+}]_o + [Na^+]_o^3 \cdot c_s$$

and where

$$K_a = \frac{1}{1 + \left(\frac{c_{naca}}{c_s}\right)^3}.$$

1.3.5 The SERCA (uptake) pump

This current pumps Ca from the bulk myoplasm into the SR and is formulated as

$$J_{up} = \frac{v_{up} c_i^2}{c_i^2 + c_{up}^2}.$$

1.3.6 The SR leak flux

The leak flux from the SR is modelled as

$$J_{leak} = g_l L(c_j)(c_j \cdot v_i / v_{sr} - c_i),$$

where $L(c_j)$ is a threshold function of the form

$$L(c_j) = \frac{c_j^2}{c_j^2 + \tilde{c}_j^2}.$$

1.3.7 Averaged Ca dynamics in the dyadic space

The average concentration in active dyadic junctions is modelled phenomenologically as

$$\frac{dc_p}{dt} = \tilde{J}_{SR} + \tilde{J}_{Ca} - \frac{c_p - c_s}{\tau_d},$$
$$\tilde{J}_{Ca} = -\tilde{g}_{Ca} P_o i_{Ca},$$
$$\tilde{J}_{SR} = -g_{SR}(V) Q\left(c_j^{\prime}\right) P_o i_{Ca},$$
$$g_{SR}(V) = \tilde{g}_{SR} \frac{e^{-0.356(V+30)}}{1 + e^{-0.356(V+30)}}.$$

1.4 Sodium dynamics

The dynamics of sodium is given by

$$\frac{d[Na^+]_i}{dt} = \frac{1}{\alpha^{\prime}} (I_{Na} + 3I_{NaCa} + 3I_{NaK}),$$

where the factor 1/α’ converts membrane currents in µA/µF to sodium fluxes in units of Mm/ms. The conversion factor is given by α’=1000α.

2 Tables

2.1 Table 1 : Ca cycling parameters

Parameter	Definition	Value
τ_r	Spark lifetime	30 ms
τ_a	NSR-JSR relaxation time	100.0 ms
g_{RyR}	Release current strength	1.22×10^{3} sparks/μM
u	Release slope	11.3 ms^{-1}
c_{sr}	Threshold for steep release function	90 M/l cytosol
b	Gain function parameter	0.05 $(mV)^{-1}$
s	Release function parameter	$(1-u)c_{sr}-50$ μM/ms
τ_d	Diffusion time constant	4 ms

2.2 Table 2 : Ca buffer parameters

Parameter	Definition	Value
B_T	Total concentration of Troponin C	70 μmol/l cytosol
B_{SR}	Total concentration of SR binding sites	47 μmol/l cytosol
B_{Cd}	Total concentration of Calmodulin binding sites	24 μmol/l cytosol
k_{on}^{T}	On rate for Troponin C binding	0.0327 $(\mu M)^{-1}(ms)^{-1}$
k_{off}^{T}	Off rate for Troponin C binding	0.0196 ms^{-1}
K_{SR}	Dissociation constant for SR binding sites	0.6 μM
K_{Cd}	Dissociation constant for Calmodulin binding sites	7 μM

2.3 Table 3: Exchanger, uptake, and SR leak parameters

Parameter	Definition	Value
c_{up}	Uptake threshold	0.5 μM
v_{up}	Strength of Uptake	0.4 μM/ms
g_{NaCa}	Strength of exchange current	0.84 μM/s
k_{sat}	Constant	0.2
ξ	Constant	0.35
$K_{m,Nai}$	Constant	12.3 mM
$K_{m,Nao}$	Constant	87.5 mM
$K_{m,Cai}$	Constant	0.0036 mM
$K_{m,Cao}$	Constant	1.3 mM
c_{naca}	Constant	0.3μM
g_l	Strength of leak current	2.069×10^{-5} ms^{-1}
$\tilde{c}_j$	Threshold for leak onset	50 μM

2.4 Table 4 : L-type Ca current parameters

Parameter	Definition	Value
g_{Ca}	Strength of Ca current flux	1.82×10^{2} mMl $ms^{-1}cm^{-1}$
$\tilde{g}_{Ca}$	Strength of local Ca flux due to L-type Ca channels	4.86 mMl $(ms\ Cm)^{-1}$
$\tilde{g}_{SR}$	Strength of local Ca flux due to RyR channels	12.7 mMl $(ms\ Cm)^{-1}$
$\tilde{c}_p$	Threshold for Ca induced inactivation	3.0 μM
$\bar{c}_p$	Threshold for Ca induced inactivation	10.0 μM
τ_{po}	Time constant of activation	1.0 ms
r_1	Opening rate	0.3 ms^{-1}
r_2	Closing rate	3 ms^{-1}
s_1'	Inactivation rate	0.00195 ms^{-1}
k_1'	Inactivation rate	0.00413 ms^{-1}
k_2	Inactivation rate	1.03615×10^{-4} ms^{-1}
k_{21}'	Inactivation rate	0.00224 ms^{-1}
T_{Ba}	Time constant	450 ms

2.5 Table 5 : Physical constants and ionic concentrations

Parameter	Definition	Value
C_m	Cell capacitance	3.1×10^{-4} μF
v_i	Cell volume	2.58×10^{-5} μl
v_s	Submembrane volume	0.02 v_i
F	Faraday constant	96.5 C/mmol
R	Universal gas constant	8.315 $Jmol^{-1}K^{-1}$
T	Temperature	308 K

$[Na^{+}]_{o}$	External sodium concentration	136 mM
$[K^{+}]_{i}$	Internal potassium concentration	140 mM
$[K^{+}]_{o}$	External potassium concentration	5.4 mM
$[Ca^{2+}]_{o}$	External calcium concentration	1.8 mM

2.6 Table 6 : Ionic current conductance

Parameter	Definition	Value
g_{Na}	Peak I_{Na} conductance	12.0 mS/μF
$g_{to,f}$	Peak $I_{to,f}$ conductance	0.11 mS/μF
$g_{to,s}$	Peak $I_{to,s}$ conductance	0.04 mS/μF
g_{K1}	Peak I_{K1} conductance	0.3 mS/μF
g_{Kr}	Peak I_{Kr} conductance	0.0125 mS/μF
g_{Ks}	Peak I_{Ks} conductance	0.1386 mS/μF
g_{NaK}	Peak I_{NaK} conductance	1.5 mS/μF

3 References

1. Mahajan A, Shiferaw Y, Sato D, Baher A, Olcese R, Xie LH, Yang MJ, Chen PS, Restrepo JG, Karma A, Garfinkel A, Qu Z, Weiss JN. A rabbit ventricular action potential model replicating cardiac dynamics at rapid heart rates. Biophysical journal. 2008;94(2):392-410. Epub 2007/12/28. doi: 10.1529/biophysj.106.98160. PubMed PMID: 18160660; PMCID: PMC2157228.

2. Shannon TR, Wang F, Puglisi J, Weber C, Bers DM. A mathematical treatment of integrated Ca dynamics within the ventricular myocyte. Biophys J. 2004;87(5):3351-71. PubMed PMID: 15347581.

3. Shiferaw Y, Watanabe MA, Garfinkel A, Weiss JN, Karma A. Model of intracellular calcium cycling in ventricular myocytes. Biophys J. 2003;85(6):3666-86. PubMed PMID: 14645059.

4. Luo CH, Rudy Y. A dynamic model of the cardiac ventricular action potential. I. Simulations of ionic currents and concentration changes. Circ Res. 1994;74(6):1071-96. PubMed PMID: 7514509.

5. Puglisi JL, Bers DM. LabHEART: an interactive computer model of rabbit ventricular myocyte ion channels and Ca transport. Am J Physiol Cell Physiol. 2001;281(6):C2049-60. PubMed PMID: 11698264.

6. Shannon TR, Ginsburg KS, Bers DM. Reverse mode of the sarcoplasmic reticulum calcium pump and load-dependent cytosolic calcium decline in voltage-clamped cardiac ventricular myocytes. Biophys J. 2000;78(1):322-33. PubMed PMID: 10620296.

7. Hund TJ, Rudy Y. Rate dependence and regulation of action potential and calcium transient in a canine cardiac ventricular cell model. Circulation. 2004;110(20):3168-74. PubMed PMID: 15505083.
